# Spanish vs USA cohort comparison of prehospital trauma scores to predict short-term mortality

**DOI:** 10.1016/j.clinme.2024.100208

**Published:** 2024-04-21

**Authors:** Diego Moreno-Blanco, Erik Alonso, Ancor Sanz-García, Elisabete Aramendi, Raúl López-Izquierdo, Rubén Perez García, Carlos del Pozo Vegas, Francisco Martín-Rodríguez

**Affiliations:** a Department of Communications Engineering, University of the Basque Country (UPV/EHU), Bilbao, Spain; b Biomedical Engineering and Telemedicine Centre, ETSI de Telecomunicación, Center for Biomedical Technology, Universidad Politécnica de Madrid, Madrid, Spain; c Department of Applied Mathematics, University of the Basque Country (UPV/EHU), Bilbao, Spain; d Faculty of Health Sciences, University of Castilla – La Mancha (UCLM), Talavera, Spain; e Faculty of Medicine, University of Valladolid, Valladolid, Spain; f CIBER of Respiratory Diseases, Instituto de Salud Carlos III, Madrid, Spain; g Emergency Department. Hospital Universitario Rio Hortega. Valladolid, Spain; h Emergency Department. Hospital Clínico Universitario. Valladolid, Spain; i Advanced Life Support, Emergency Medical Services (SACYL), Valladolid, Spain

**Keywords:** Prehospital, Trauma, Early warning scores, Short-term mortality

## Abstract

**Background:**

This study aimed to evaluate three prehospital early warning scores (EWSs): RTS, MGAP and MREMS, to predict short-term mortality in acute life-threatening trauma and injury/illness by comparing United States (US) and Spanish cohorts.

**Methods:**

A total of 8,854 patients, 8,598/256 survivors/nonsurvivors, comprised the unified cohort. Datasets were randomly divided into training and test sets. Training sets were used to analyse the discriminative power of the scores in terms of the area under the curve (AUC), and the score performance was assessed in the test set in terms of sensitivity (SE), specificity (SP), accuracy (ACC) and balanced accuracy (BAC).

**Results:**

The three scores showed great discriminative power with AUCs>0.90, and no significant differences between cohorts were found. In the test set, RTS/MREMS/MGAP showed SE/SP/ACC/BAC values of 86.0/89.9/89.6/87.1%, 91.0/86.9/87.5/88.5%, and 87.7/82.9/83.4/85.2%, respectively.

**Conclusions:**

All EWSs showed excellent ability to predict the risk of short-term mortality, independent of the country.

## Introduction

Emergency medical services (EMS) routinely handle acute life-threatening trauma and injuries/illnesses quickly and accurately. In potentially life-threatening conditions, EMS providers conduct initial assessments based on systematic and systematised X-A-B-C-D-E protocols.^1^ The critical challenge on-scene or en route is to detect high-risk patients without obvious clinical manifestations to carry out a highest-priority referral to the emergency department (ED) and provide follow-up care.^2^

Trauma patients present several challenges in prehospital care, including potentially hazardous scenarios, ie traffic accidents, fires, explosions, which result in trouble fulfilling the medical history, limited complementary tests, joint operations involving different first responders on scene, or sometimes even with opposing strategies and priorities.^3^ In this regard, on-scene early warning scores (EWSs) have been demonstrated to be a promising and effective way to quickly detect high-risk cases. EWS comprising vital signs, eg respiratory rate, oxygen saturation, systolic blood pressure, heart rate and level of consciousness, can be handled by staff with basic training and provide reliable predictive performance^4^

Clinical application of EWS is a reality in intensive care units (ICUs) or EDs, but with tentative implantation in prehospital care.^5^ In trauma patients, scoring systems have been a well-established practice. The original trauma score was developed by Champion HR *et al*^6^ in 1981 and updated in 1989^7^ to the revised trauma score (RTS). In parallel to RTS, the Glasgow Coma Scale, created by Teasdale & Jennett in 1974,^8^ is internationally accepted and included in all guidelines for the initial evaluation and management of acute life-threatening trauma and injuries/illnesses, with particular relevance to the sequential assessment of traumatic brain injury.^9^

Accidents involving trauma and injury are complex scenarios, often with multiple victims on-scene. Under these circumstances, the EWS can help decisively perform an efficient, quick, and reliable first triage,^10^ as shown by RTS.^11^ EWS or trauma scores play a key role in triggering early identification and tripping of trauma codes and subsequent high-priority evacuation to trauma centres.^12^

The goal of the present study was to evaluate three prehospital EWSs to predict short-term mortality in acute life-threatening trauma and injury/illness transferred in ambulances to trauma centres, comparing US and Spanish cohorts.

## Methods

### Study design

This was a multicentre, EMS-based, observational study involving a prospective dataset, ‘Prehospital Identification of Prognostic Biomarkers in Time-dependent Diseases’ (HITS), and a retrospective dataset, ‘National Emergency Medical Services Information System’ (NEMSIS).^13^

The institutional review board of the Public Health Service approved the study (# PI041-19, # PI217-20). The institutional research granted a waiver/exemption for NEMSIS owing to the use of deidentified data. We followed the transparent reporting of a multivariable prediction model for individual prognosis or diagnosis (TRIPOD)^14^ guidelines.

### Study settings

The HITS study collected prospective data between 1 January 2020 and 31 December 2022 in four Spanish provinces (Burgos, Salamanca, Segovia and Valladolid). EMS is operated by the Public Health System and is integrated by Advance Life Support (staffed by two emergency medical technicians, an emergency registered nurse and a physician), Helicopter Emergency Medical Service (staffed by an emergency registered nurse and a physician) and Basic Life Support (staffed by two emergency medical technicians).

NEMSIS included retrospective data between 1 January and 31 December 2017, a nationally representative dataset of EMS activations populated by more than twelve thousand EMS agencies throughout the US. The EMS included in the dataset is integrated by Advance Life Support (staffed by for two paramedics), Helicopter Emergency Medical Service (staffed by an emergency registered nurse and/or a physician) and Basic Life Support (staffed by two emergency medical technicians).

### Population

All consecutive adult patients (>18 years) with trauma and injury diseases who were evacuated with high priority to emergency trauma centres were included in the analysis. Minors and all cases involving missing data impossible to impute were excluded (details in the data management section).

The trauma code was activated in the following cases: amputation proximal to wrist or ankle, crushed, degloved, mangled, or pulseless extremity, chest wall instability or deformity (eg flail chest), Glasgow Coma Score (GCS) ≤ 13, open or depressed skull fracture, paralysis, pelvic fractures, all penetrating injuries to head, neck, torso, and extremities proximal to elbow or knee, respiratory rate <10 or >29 breaths per min or need for ventilatory support, systolic blood pressure <90 mmHg, two or more proximal long-bone fractures, burns (scalds)/explosions and carbon monoxide inhalation.

### Outcome

The primary outcome was short-term mortality. For NEMSIS, short-term mortality was extrapolated from ED and hospital disposition, and for HITS, 2-day mortality (all-cause and in- and out-of-hospital) obtained from the electronic health record was considered.

### Score selection

The EWS used in this study were selected by considering (i) their feasibility in the prehospital setting, (ii) with no analytical parameters, (iii) not including temperature as a variable (since it was not available in the NEMSIS), and (iv) scoring systems already validated for use in trauma patients. Therefore, the selected scores included RTS,^7^ Mechanism/GCS/Age/Pressure score (MGAP)^15^ and Modified Rapid Emergency Medicine Score (MREMS).^16^ Supplementary Table 1 shows in detail the parameters comprising each score.

### Data management

**Fig. 1:**
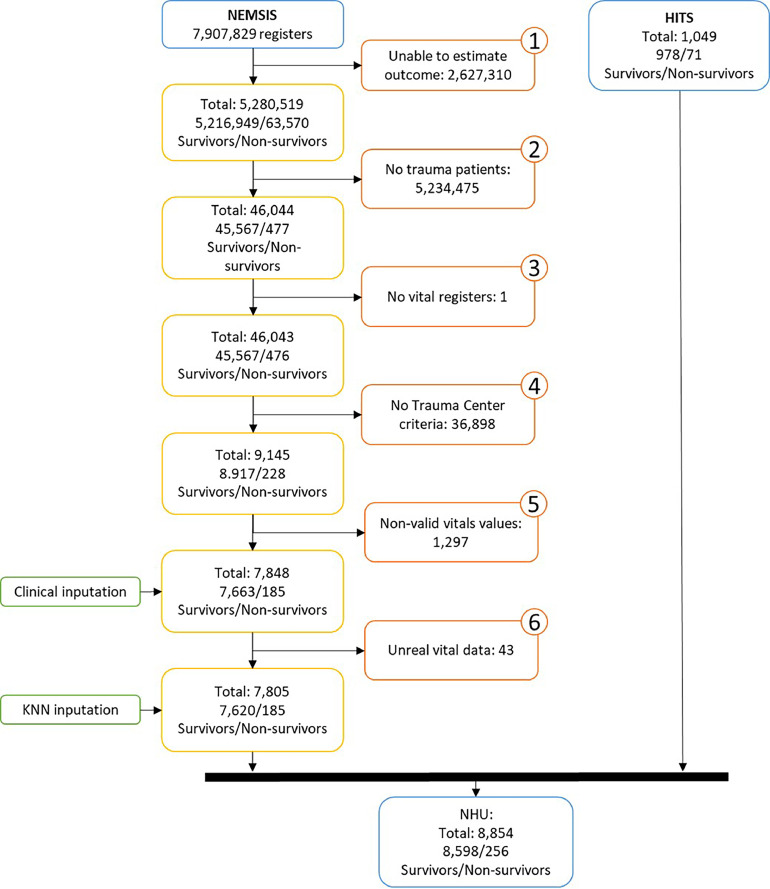
Flowchart of the HITS and NEMSIS datasets. Including data management and outcome imputation for the NEMSIS dataset. *Abbreviations*: NEMSIS: National Emergency Medical Services Information System; HITS: Prehospital Identification of Prognostic Biomarkers in Time-dependent Diseases; NHU: NEMSIS-HITS Unified.

All prehospital information was collected directly by the EMS providers during the first contact with the patient, filling out the corresponding clinical-assistance reports. The NEMSIS dataset provides two fields to identify the final outcome of each patient: ED disposition and hospital disposition. Of the 7,907,829 cases, only 161,348 (2.04%) had at least one of the disposition elements filled. Fig. 1 shows the flowchart of the process followed to overcome this problem. First, the end-of-event outcome indicator proposed by Miller *et al* for the NEMSIS dataset was applied.^17^ Briefly, the end-of-event outcome indicator consists of validated criteria based on alternative elements and codes in the NEMSIS dataset, providing information about a patient's end-of-event status. Thus, the outcome of a total of 5,280,519 cases was identified, 5,216,949 survivors and 63,570 nonsurvivors, while 2,627,310 were discarded due to lack of outcome. Second, traumatic adult patients were selected, resulting in a total of 46,044 cases. Third, a single case with no vital sign recordings was discarded. Fourth, 36,898 cases were excluded due to unavailability of the Trauma Centre Criteria. In the fifth step, cases lacking at least three out of the four vitals recorded (respiratory rate, oxygen saturation, heart rate and systolic blood pressure) were discarded. The last step consisted of vital sign imputation in those cases when at most two vital signs were missing. For that purpose, two methods were consecutively applied. First, a clinical imputation method (thoroughly described in the supplementary materials) was used, after which 43 cases with systolic pressures above 250 mmHg and respiratory rates above 60 breaths per min were considered unrealistic and consequently removed (step 6 in Fig. 1). Then, a machine learning method, the *K*-nearest neighbours (KNN),^18^ was applied with *K*=10 to search for the *K* cases with the most similar vitals (‘neighbours’) to those present in the case to be imputed. Thus, the method completed the missing vitals using the average of the vitals of those *K*neighbours identified (see supplementary materials). Therefore, the final NEMSIS dataset for this study contained a total of 7,805 cases.

The HITS dataset (*n* = 1,049 cases) did not present missing values, so no imputation methods or other data transformation techniques were applied. Both datasets were merged into the NEMSIS-HITS Unified (NHU) dataset with a total of 8,854 cases.

### Data analysis

Quantitative variables were expressed as the median (interquartile range, IQR), as they did not pass the Kolmogorov‒Smirnov normality test. Qualitative variables were expressed as absolute values and percentages, *N* (%). For comparing quantitative/qualitative variables in the survivor and nonsurvivor groups, the Mann–Whitney U test/Chi-square test was performed to test for equal medians/no significant differences, respectively. A *p* value < 0.05 was considered statistically significant.

The datasets (NEMSIS, HITS and NHU) were randomly divided into training (70%) and test (30%) quasistratified sets. Each set maintained a minimum of 90% of the survivor and nonsurvivor proportions. The training set was used to assess the discriminative power of the scores for distinguishing between survivors and nonsurvivors separately for each dataset. The evaluation was carried out in terms of receiver operating characteristic (ROC) curves and their associated metrics, namely, the area under the curve (AUC), optimum decision threshold that maximised the Youden index, sensitivity (SE, capacity to correctly detect nonsurvivors), specificity (SP, capacity to correctly detect survivors), accuracy (ACC, capacity to correctly detect both survivors and nonsurvivors) and balanced accuracy (BAC, mean value of SE and SP) together with their 95% confidence interval (CI). Furthermore, for each ROC curve,^1^ the 95% CI was computed using 1,000 stratified bootstraps, and^2^ the p value of the comparison against chance levels (ROC curve with AUC = 0.5) was determined. Delong's test was used to test for differences in the AUC of the ROC curves for the NEMSIS and HITS datasets.

The test set was used to assess the performance of each score using the optimum decision threshold identified in the training set. The assessment was also carried out in terms of AUC, SE, SP, ACC, and BAC.

All data processing and statistical analyses were performed in Python, version 3.11 (https://www.python.org/), using our own codes.

## Results

A total of 7,805 cases from the NEMSIS and 1,049 cases from the HITS datasets fulfilled the inclusion criteria (a total of 8,854 for NHU) (see Fig. 1).

A total of 97.1% (8,598) were survivors, with 27.7% being females (2,378) and a median age of 42 (28–59) years, with the 18–49 years age group standing out (60.9 %). The mortality rate was 2.9% (256), with 24.2% females and a median age of 45 (30–64) years, also highlighting the 18–49 years group in the nonsurvivors. All the EWSs analysed, RTS, MGAP and MREMS, showed significant differences between survivors and nonsurvivors (*p* < 0.001). Primary trauma code activations in nonsurvivors were GCS≤13 points, all penetrating injuries to the head, neck, torso, and extremities proximal to the elbow or knee, and respiratory rate <10 or >29 breaths per min or need for ventilatory support (Table 1).

**Table 1:** Baseline patient characteristics according to short-term mortality in the NHU dataset.

Variable^b^	Short-term mortality		*p* value	Odds ratio (95 %CI)
	Survivor^a^	Nonsurvivor^a^		
No. (%) with data	8,598 (97.1)	256 (2.9)	NA	NA
**Epidemiological variables**				
Sex, female	2,378 (27.7)	62 (24.2)	0.435	0.879 (0.638, 1.214)
Age, year	42 (28–59)	45 (30–64)	0.958	1.000 (0.999, 1.000)
Age groups, year				
18–49	5,239 (60.9)	145 (56.6)		
50–74	2,440 (28.4)	68 (26.6)	0.963	1.007 (1.194, 2.393)
≥ 75	919 (10.7)	43 (16.8)	0.003	1.691 (1.194, 2.393)
Penetrating trauma	1,486 (17.3)	34 (13.3)	0.428	0.853 (0.576, 1.263)
**On-scene vital signs**				
RR, breaths/min	18 (16–20)	8 (0–18)	<0.001	0.799 (0.781, 0.817)
Oxygen saturation, %	97 (95–99)	86 (0–95)	<0.001	0.947 (0.941, 0.952)
SBP, mmHg	132 (114–148)	70 (0–123)	<0.001	0.963 (0.959, 0.965)
Heart rate, beats/min	92 (80–109)	54 (0–104)	<0.001	0.961 (0.957, 0.965)
Glasgow Coma Scale, points	15 813–15)	3 (3–5)	<0.001	0.715 (0.690, 0.740)
**Trauma code activation conditions**				
Amputation proximal to wrist or ankle	471 (5.5)	13 (5.1)		
Crushed, degloved, mangled, or pulseless extremity	442 (5.1)	14 (5.5)	0.725	1.148 (0.534, 2.469)
Chest wall instability or deformity	317 (3.7)	9 (3.5)	0.949	1.029 (0.435, 2.435)
Glasgow Coma Score ≤ 13	3,577 (41.6)	137 (53.5)	0.266	1.388 (0.779, 2.471)
Open or depressed skull fracture	389 (4.5)	12 (4.7)	0.784	1.118 (0.504, 2.478)
Paralysis	203 (2.4)	1 (0.4)	0.098	0.179 (0.023, 1.374)
Pelvic fractures	390 (4.5)	7 (2.7)	0.364	0.650 (0.257, 1.646)
All penetrating injuries	1,450 (16.9)	25 (9.8)	0.174	0.625 (0.317, 1.231)
RR <10 or >29 breaths per or need for ventilatory support	440 (5.1)	25 (9.8)	0.038	2.059 (1.040, 4.074)
Systolic blood pressure <90 mmHg	252 (2.9)	7 (2.7)	0.989	1.006 (0.397, 2.555)
Two or more proximal long-bone fractures	652 (7.6)	3 (1.2)	0.005	0.167 (0.047, 0.588)
Burns (scalds)/explosions and carbon monoxide inhalation	15 (0.2)	3 (1.2)	0.004	7.247 (1.867, 28.139)
**Scores calculation**				
Revised trauma score	12 (11–12)	4 (1–8)	<0.001	0.491
MGAP	25 (22–28)	12 (12–17)	<0.001	0.712
MREMS	3 (1–5)	15 (11–21)	<0.001	1.537

*Abbreviations*: CI: confidence interval; NA: not applicable; RR: respiratory rate; SBP: systolic blood pressure; MGAP: Mechanism/Glasgow Coma Scale/Age/Pressure score; MREMS: Modified Rapid Emergency Medicine Score; OR: odds ratio.

a Values expressed as total number (percentage) and median (IQR), as appropriate.

An isolated analysis of NEMSIS and HITS showed a net difference in mortality (2.5% vs. 6.8%). The median age of nonsurvivors in the NEMSIS was 41 (27–55) years, with 63.8% (118) distributed at 18–49 years of age. The reasons for activation of the trauma code were GCS≤13 points and all penetrating injuries to the head, neck, torso, and extremities proximal to the elbow or knee. In contrast, the median age in HITS was 62 (40–81) years, with the majority of nonsurvivors in the 50–74 years age group (40.8%), highlighting trauma code activations by GCS≤13 points and systolic blood pressure <90 mmHg (Table 2).

**Table 2:** Baseline patient characteristics according to cross-cohort comparison.

Variable^b^	NEMSIS		*p* value^b^	HITS		*p* value^b^
	Survivor^a^	Nonsurvivor^a^		Survivor^a^	Nonsurvivor^a^	
No. (%) with data	7,620 (97.6)	185 (2.5)	NA	978 (93.2)	71 (6.8)	NA
**Epidemiological variables**						
Sex, female	2,025 (26.6)	39 (21.1)	0.094	353 (36.1)	23 (32.4)	0.531
Age, year	40 (27–58)	41 (27–55)	0.697	52 (37–70)	62 (40–81)	0.040
Age groups, year						
18–49	4,801 (63)	118 (63.8)	0.577	438 (44.8)	27 (38)	0.003
50–74	2,092 (27.5)	53 (28.9)		348 (35.6)	15 (21.1)	
≥ 75	729 (9.5)	14 (7.6)		192 (19.6)	29 (40.8)	
Penetrating trauma	1,433 (18.8)	25 (13.5)	0.068	53 (5.4)	9 (12.7)	0.012
**Trauma code activation conditions**						
Amputation proximal to wrist or ankle	467 (6.1)	13 (5.9)	0.156	4 (0.4)	0	<0.001
Crushed, degloved, mangled, or pulseless extremity	438 (5.7)	12 (6.5)		4 (0.4)	2 (2.8)	
Chest wall instability or deformity	216 (2.8)	9 (4.9)		101 (10.3)	0	
Glasgow Coma Scale ≤ 13	3,445 (45.2)	85 (45.9)		131 (13.4)	52 (73.2)	
Open or depressed skull fracture	166 (2.2)	11 (5.9)		223 (22.8)	1 (1.4)	
Paralysis	155 (2)	0		48 (4.9)	1 (1.4)	
Pelvic fractures	340 (4.5)	2 (1.1)		50 (5.1)	5 (7.4)	
All penetrating injuries	1,433 (18.8)	25 (13.5)		17 (1.7)	0	
RR <10 or >29 breaths per or need for ventilatory support	408 (5.4)	23 (12.4)		32 (3.3)	2 (2.8)	
Systolic blood pressure <90 mmHg	216 (2.8)	2 (1.1)		36 (3.7)	5 (7.4)	
Two or more proximal long-bone fractures	335 (4.4)	3 (1.6)		317 (32.4)	0	
Burns (scalds)/explosions and carbon monoxide inhalation	0	0		15 (1.5)	3 (4.2)	
**Scores calculation**						
Revised trauma score	12 (11–12)	4 (0–6)	<0.001	12 (12–12)	8 (6–10)	<0.001
MGAP	25 (22–27)	12 (12–16)	<0.001	27 (24–29)	17 (13–19)	0.001
MREMS	3 (1–5)	16 (12–22)	<0.001	3 (1–5)	12 (9–16)	<0.001

*Abbreviations*: CI: confidence interval; NA: not applicable; MGAP: Mechanism/Glasgow Coma Scale/Age/Pressure score; MREMS: Modified Rapid Emergency Medicine Score.

a Values expressed as total number (percentage) and medians (25th–75th percentile), as appropriate.

b The Mann‒Whitney U test or chi-squared test was used as appropriate.

**Fig. 2:**
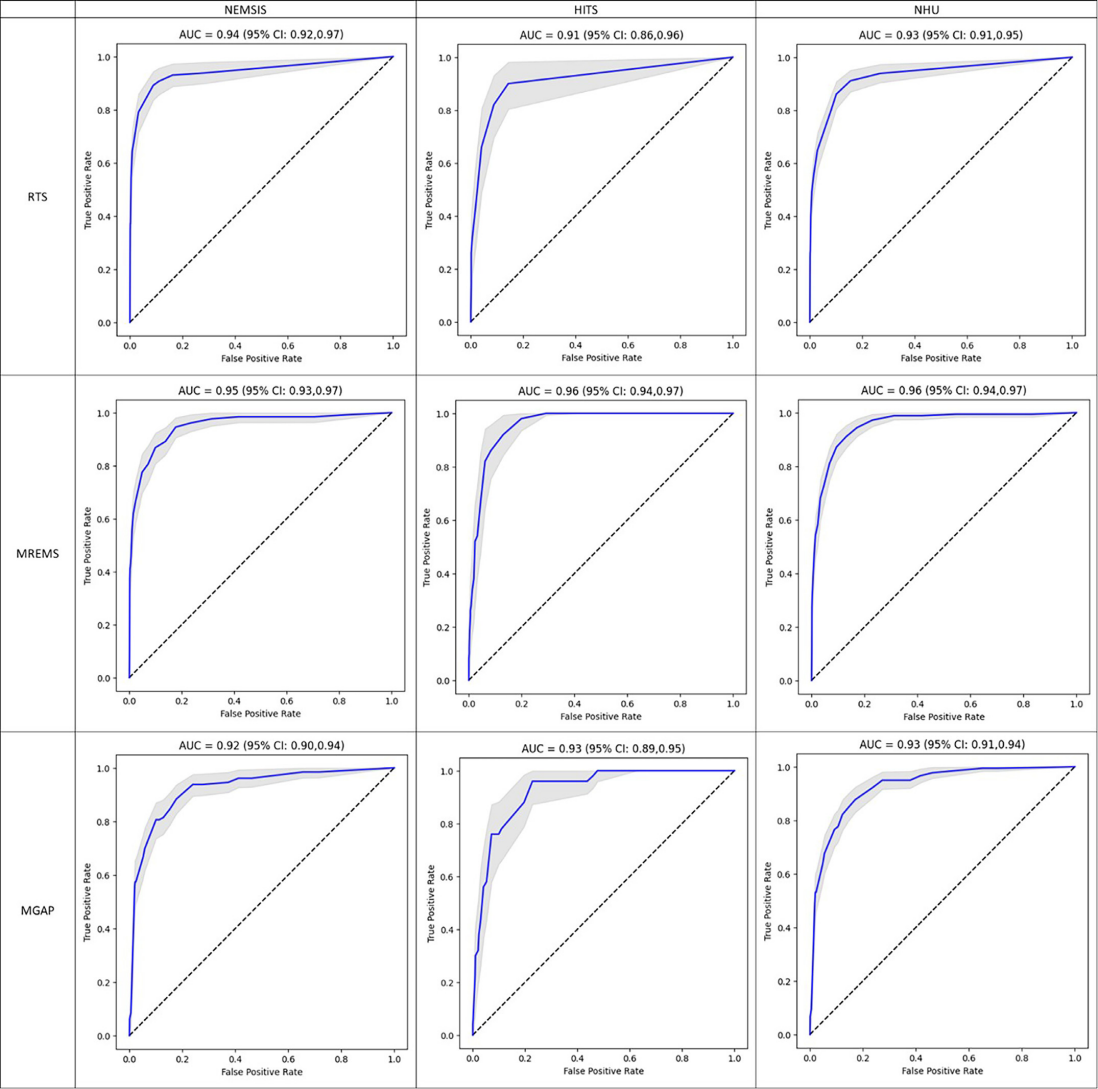
ROC curves (blue) and their 95% CI (shadowed in gray) for each score computed in the training set of the NEMSIS, HITS and NHU datasets from left to right. *Abbreviations*: NEMSIS: National Emergency Medical Services Information System; HITS: Prehospital Identification of Prognostic Biomarkers in Time-dependent Diseases; NHU: NEMSIS-HITS Unified; RTS: Revised Trauma Score; MREMS: Modified Rapid Emergency Medicine Score; MGAP: Mechanism/Glasgow Coma Scale/Age/Pressure score.(For interpretation of the references to colour in this figure legend, the reader is referred to the web version of this article.)

The performance of each score in the training set of the three datasets is described in Fig. 2 and Table 3. Fig. 2 shows the ROC curves (95% CI shadowed in gray), and Table 3 summarises the performance metrics. Delong's test reported no differences (*p* value > 0.05) between the AUCs obtained by the scores for the NEMSIS and HITS datasets, which supports the merging of both datasets into the NHU. The three scores showed great discriminative power for distinguishing between survivors/nonsurvivors, with AUCs above 0.90 and BACs above 85.2% in any dataset. Specifically, the RTS/MREMS/MGAP showed AUCs (95% CI) of 0.93 (0.91–0.95), 0.96 (0.94–0.97), and 0.93 (0.91–0.94), respectively, in the NHU dataset.

**Table 3:** Discriminative power of the scores in terms of AUC in the training set of the NEMSIS, HITS and NHU datasets.

**Score**	Dataset	AUC^a^	OPT. TH**^a^**	SE (%)^a^	SP (%)^a^	ACC (%)^a^	BAC (%)^a^	*p* value^b^
**RTS**	NEMSIS	0.94 (0.92–0.97)	7 (6–8)	89.1 (84.2–95.2)	91.1 (88.2–96.5)	91.0 (89.2–97.1)	92.8 (90.7–92.8)	0.3001
	HITS	0.91 (0.86–0.96)	10 (9–10)	90.0 (78.4–96.7)	85.7 (83.5–92.9)	85.7 (85.7–90.1)	87.9 (85.9–87.9)	
	NHU	0.93 (0.91–0.95)	8 (8–9)	86.0 (82.8–94.3)	89.9 (83.8–90.6)	89.6 (84.7–89.6)	87.1 (87.1–87.1)	
**MREMS**	NEMSIS	0.95 (0.93–0.97)	9 (7–10)	86.8 (83.8–97.6)	90.0 (81.5–92.7)	89.9 (82.1–92.6)	91.3 (90.0–91.5)	0.6452
	HITS	0.96 (0.94–0.97)	7 (6–9)	92.0 (84.9–100)	86.9 (78.2–94.4)	92.1 (85.1–95.2)	95.3 (86.4–95.2)	
	NHU	0.96 (0.94–0.97)	8 (7–9)	91.0 (85.6–96.6)	86.9 (82.3–91.2)	87.5 (83.3–90.2)	88.5 (87.4–89.5)	
**MGAP**	NEMSIS	0.92 (0.90–0.94)	21 (17–22)	88.4 (78.6–96.3)	82.3 (75.5–90.6)	82.5 (76.5–89.9)	88.4 (85.3–90.5)	0.8339
	HITS	0.93 (0.89–0.95)	23 (19–23)	96.0 (78.0–100)	77.2 (74.5–94.0)	77.8 (77.8–93.6)	85.8 (85.5–87.9)	
	NHU	0.93 (0.91–0.94)	21 (19–22)	87.7 (80.3–94.9)	82.9 (75.5–88.6)	83.4 (77.4–87.3)	85.2 (84.0–85.3)	

*Abbreviations*: NEMSIS: National Emergency Medical Services Information System; HITS: Prehospital Identification of Prognostic Biomarkers in Time-dependent Diseases; NHU: NEMSIS-HITS Unified; RTS: Revised Trauma Score; MREMS: Modified Rapid Emergency Medicine Score; MGAP: Mechanism/Glasgow Coma Scale/Age/Pressure score; AUC: area under the curve; OPT. TH: optimum threshold; SE: sensitivity; SP: specificity; ACC: accuracy; BAC: balanced accuracy

a Values expressed as total number (95% CI).

b *p*-value resulting from DeLongʼs test.

The optimum thresholds maximising the Youden index obtained for each score in the training set (see Table 3) were used to evaluate the performance of the scores in the test set of the NHU dataset. The RTS/MREMS/MGAP showed SE/SP/ACC/BAC values of 86.0/89.9/89.6/87.1%, 91.0/86.9/87.5/88.5%, and 87.7/82.9/83.4/85.2%, respectively.

**Fig. 3:**
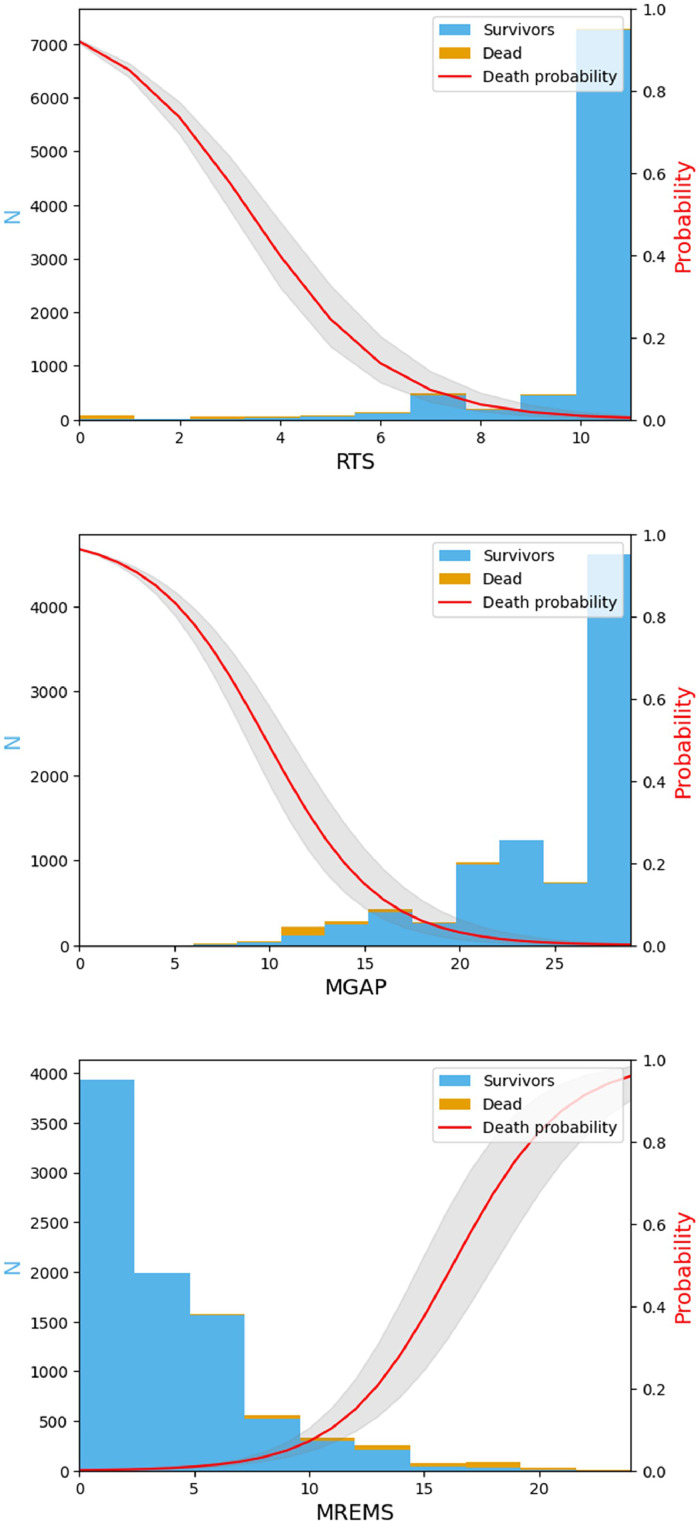
Probability of death in the NHU dataset as a function of RTS, MGAP and MREMS from top to bottom. The bar graph shows the number of patients (survivors in blue and nonsurvivors in orange). The red line reflects the estimated probability of death. *Abbreviations*: RTS: Revised Trauma Score; MREMS: Modified Rapid Emergency Medicine Score; MGAP: Mechanism/Glasgow Coma Scale/Age/Pressure score.(For interpretation of the references to colour in this figure legend, the reader is referred to the web version of this article.)

Fig. 3 shows the probability of death (red line) as a function of RTS, MGAP and MREMS for the NHU dataset. The probability of death was estimated via a logistic regression model based on each score and optimised using the training set of the NHU. As RTS and MGAP increase, the number of nonsurvivors and consequently the predicted probability of death decrease. In contrast, as MREMS increases, the number of nonsurvivors and the probability of death rise.

## Discussion

In this multicentre, EMS-based, observational study involving a prospective and retrospective dataset study, three prehospital care EWSs were compared to predict short-term mortality in 8,854 trauma patients transferred with high priority by ambulance to a trauma centre. Globally (NHU dataset), MREMS had the best predictive performance (AUC=0.96; 95 % CI: 0.94–0.97), followed by MGAP and RTS, but with no statistically significant difference between scores.

Previous studies have examined the ability of RTS^7^ to predict the risk of short-term mortality. Cassignol *et al*^3^ analysed RTS to predict in-hospital mortality with an AUC of 0.84, and Gang *et al*^19^ analysed RTS with an AUC of 0.81, performances below those obtained for the HITS, NEMSIS or NHU datasets (AUC=0.93, NHU). Sewalt *et al*^20^ compared different trauma models to identify major trauma, showing an AUC of 0.79 for MREMS, lower than the 0.96 reported in our cohort. Finally, Van Rein *et al*^21^ evaluated different prehospital triage systems in trauma patients, showing that MGAP has a predictive capacity of 0.82 (an AUC of 0.93 in the NHU dataset).

By comparing US (NEMSIS) and Spanish (HITS) cohorts, a striking discrepancy in mortality between the two populations was found: 2.5% (185) vs. 6.8% (71). This gap presumably has two key roots. First, the inclusion criteria differed. Cases in the NHU dataset were all referred to a trauma centre, with prior activation by trauma code EMS. In the NEMSIS study, inclusion criteria were less restrictive, a factor that may have influenced the mortality ratio by including minor trauma patients excluded in the HITS dataset.^22^ Second, a remarkable disparity in the median age of nonsurvivors, 41 vs. 62 years, aligns with data from comparable studies confirming demographic trends and trauma-patient epidemiology of the two regions.^12^^,^^23^^,^^24^ Furthermore, both reasons might explain the better prognostic capacity of the three EWSs analysed in our study compared to the studies cited above.^3^^,^^19^^,^^20^^,^^21^

The most common cause-specific activating trauma code in both cohorts was GCS≤13 points. The second most frequent cause of activation in the NEMSIS dataset was penetrating injuries to the head, neck, torso, and extremities proximal to the elbow or knee and respiratory rate <10 or >29 breaths per min or need for ventilatory support. For HITS, the second main causes for activation and ambulance transfer to a trauma centre were systolic blood pressure <90 mmHg and pelvic fractures. Notably, compared to the elevated number of penetrating injuries present in the NEMSIS, the HITS only presented 25 cases of penetrating injuries, none of which were fatal. In the Spanish cohort, the presence of blunt polytrauma with fatal outcomes (systolic blood pressure <90 mmHg and pelvic fractures) stands out. Evidence of these differences may be due to epidemiological and injury mechanism variability between cohorts.^25^

The use of EWS in trauma patients, except in the ICU or ED, has started quickly.^26^^,^^27^ In prehospital care, previous evidence is available using EWS to discriminate and identify high-risk patients on scene, but EWS utilisation seems to be less widespread than in other clinical scenarios.^11^^,^^28^ Acute life-threatening trauma and injuries/illnesses require quick activation of the trauma code to perform appropriate emergency support of potentially life-threatening conditions and to provide a high-priority transfer to a dedicated trauma centre. In this sense, EWSs are easy-to-use tools, requiring only a set of basic vital signs, which could aid in the on-scene decision-making process.

Several strengths emerged in the study. First, two different cohorts were evaluated, with diverse baseline targets, and in separate locations, minimising biases. In addition, all trauma patients examined were evacuated (in both groups) to a trauma centre, resulting in a final dataset that was very consistent and comparable. Therefore, this study provides reliable evidence of the generalisability of the study results. However, the study also presented some limitations. First, different variables were gathered in the two studies. Based on available variables, only those EWSs that could be calculated in both cohorts and consequently compared were selected. Nevertheless, the scorecards selected have been validated and have previous evidence in prehospital care.^7^^,^^15^^,^^16^ Second, short-term mortality was selected as the primary outcome for this study, a decision in line with other publications where the cause of mortality was directly related to trauma life-threatening conditions.^29^ Moreover, the selected primary outcome is indeed a trade-off to unify the different outcomes recorded in the HITS and NEMSIS datasets. While in the HITS, 2-day mortality was recorded and therefore used as the primary outcome, in the NEMSIS, short-term mortality had to be either extrapolated from ED and hospital disposition (only available in 2% of the cases) or imputed following the end-of-event outcome indicator developed and validated by Miller *et al*^17^ exclusively for the NEMSIS dataset. Finally, findings should be cautiously interpreted, as compared cohorts come from two distinctly divergent EMS systems, the Anglo-American paramedic and EMT system based on “scoop and run” vs. the European physician, emergency registered nurse and ETM system based on 'stay and play'.^30^ Nevertheless, the results of all EWS, performed by different cohorts or in the unified dataset, are consistent.

In summary, although comparing datasets from EMS systems with different workflows, with notable variations in median age, and with diverse biomechanics involved in the injuries, all EWSs examined showed an excellent ability to predict the risk of short-term mortality in trauma patients. Therefore, the implementation of EWS for use by on-scene EMS providers should be an emerging worldwide trend in prehospital care.

**Summary box**

**What is known**

Early warning scores (EWSs) have been demonstrated to be a promising and effective way to quickly detect high-risk cases.

**What is the question**

This study aimed to evaluate three prehospital early warning scores (EWS) in acute life-threatening trauma in tow cohorts from US and Spain.

**What was found**

All EWS showed excellent ability to predict the risk of mortality, independent of country.

**What is the implication for practice now?**

The use of EWS by emergency medical services should be implemented worldwide.

Alt-text: Unlabelled box

## Summarised conflict of interest statements

All signing authors meet the requirements of authorship and have declared no conflicts of interest. The authors have no disclosures to make. On behalf of the other authors, the corresponding author guarantees the accuracy, transparency and honesty of the data and information contained in the study, that no relevant information has been omitted and that all discrepancies between authors have been adequately resolved and described.

## Funding information

This work was partially supported by MCIN/AEI/10.13039/501100011033 and “ERDF A way of making Europe” under grant PID2021-122727OB-I00; by the Basque Government under grant IT-1717-22 and by the 10.13039/501100003451University of the Basque Country (UPV/EHU) under grant COLAB20/01. This work was also supported by the Gerencia Regional de Salud, Public Health System of Castilla y León (Spain) [grant number GRS 1903/A/19 and GRS 2131/A/20]. CIBER de Enfermedades Respiratorias, Instituto de Salud Carlos III [grantnumber CB22/06/00035].

## Ethics approval

The study was approved by the local institutional research review board of Public Health Service. The institutional research granted a waiver/exemption for the NEMSIS dataset owing to the use of deidentified data.

## Notation of prior abstract publication/presentation

This article is an original work, has not been published before and is not being considered for publication elsewhere in its final form, in either printed or electronic media. It is not based on any previous communication to a society or meeting.

## Availability of data and materials

The datasets used and/or analysed during the current study are available from the corresponding author on reasonable request.

## CRediT authorship contribution statement

**Diego Moreno-Blanco:** Data curation, Methodology, Visualization, Software, Validation, Writing – original draft, Writing – review & editing. **Erik Alonso:** Data curation, Methodology, Visualization, Software, Validation, Writing – original draft, Writing – review & editing. **Ancor Sanz-García:** Data curation, Methodology, Visualization, Software, Validation, Writing – original draft, Writing – review & editing. **Elisabete Aramendi:** Supervision, Methodology, Software, Investigation, Writing – review & editing. **Raúl López-Izquierdo:** Supervision, Methodology, Software, Investigation, Writing – review & editing. **Rubén Perez García:** Supervision, Methodology, Software, Investigation, Writing – review & editing. **Carlos del Pozo Vegas:** Supervision, Methodology, Software, Investigation, Writing – review & editing. **Francisco Martín-Rodríguez:** Conceptualization, Data curation, Supervision, Writing – original draft, Writing – review & editing, Methodology, Investigation.

## Appendix Supplementary materials

Image, application 1
