# Supplementary material for: Spanish vs USA cohort comparison of prehospital trauma scores to predict short-term mortality
Source: Clin Med (Lond). 2024 Apr 21;24(3):100208. doi: 10.1016/j.clinme.2024.100208 (PMC11101846; doi:10.1016/j.clinme.2024.100208)
Supplement: Supplementary file 1 [file mmc1.docx]

**Supplementary material**


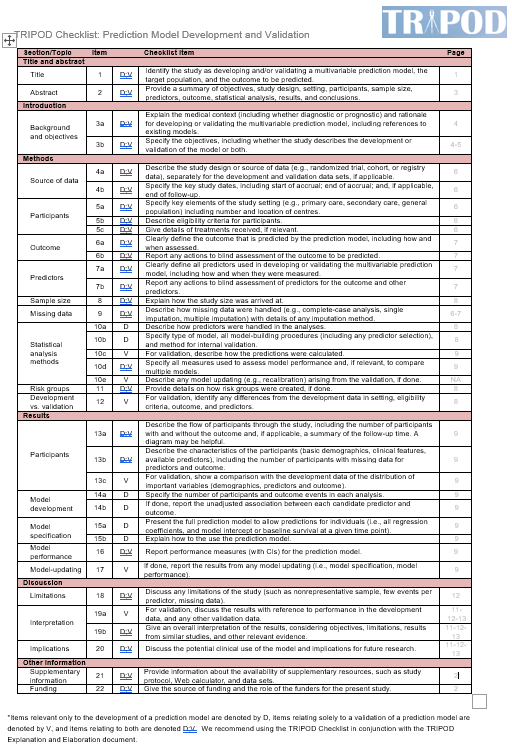


Supplementary Table 1 summarizes the variables considered in each one of the early warning scores (EWS) computed and compared in the study. Those variables are the respiratory rate, oxygen saturation, systolic blood pressure, heart rate, Glasgow coma scale, age and trauma mechanism, which are easy to acquire in a prehospital setting.

Supplementary Table 1. Prehospital early warning scores evaluated, and variables made up.

| Variable | RTS | MGAP | MREMS |
| --- | --- | --- | --- |
| Respiratory rate | X |  | X |
| Oxygen saturation |  |  | X |
| Systolic blood pressure | X | X | X |
| Heart rate |  |  | X |
| Glasgow coma scale | X | X | X |
| Age |  | X | X |
| Trauma mechanism |  | X |  |

*Abbreviations:* RTS: Revised trauma score; MGAP: Mechanism/Glasgow Coma Scale/Age/Pressure score; MREMS: Modified Rapid Emergency Medicine Score

**Clinical imputation**

The first method used for vital sign and/or Glasgow Coma Score (GCS) imputation in the NEMSIS dataset was based on clinical criteria and was applied in those cases when GCS and/or at most two vitals were missing. The four vitals are Systolic Blood Pressure (SBP), Heart Rate (HR), Respiratory Rate (RR) and Oxygen Saturation (SpO2). Other fields in NEMSIS dataset provide complementary information on these fields, allowing us to estimate an approximate value of the missing variable. Particular focus has been put on the Trauma Center Criteria (TCC) and level of responsiveness (variable AVPU standing for Alert, Verbal, Painful, Responsiveness) fields. The TCC can contain some codes that specify a range of possible values for vitals while AVPU values can be transformed into a GCS. Other available vitals were also taken into account for imputing the missing ones. The method applies the following sequence for each of the five parameters described:

1. SBP was imputed following the flowchart presented in Supplementary Figure 1. First, if the heart is not contracting then SBP should be set to zero. So, if heart rate was 0 beats per minute (bpm), SBP was imputed to 0 mmHg. TCC can contain a code that defines SBP below 90 mmHg. In that case SBP was filled with a value of 80 mmHg. This value was used because all the scorecards analyzed give the same score to any value under 90 mmHg. Also if at least two of the other vitals analyzed were zero, then SBP was imputed to 0 mmHg. If none of these conditions were satisfied, SBP remained missing.


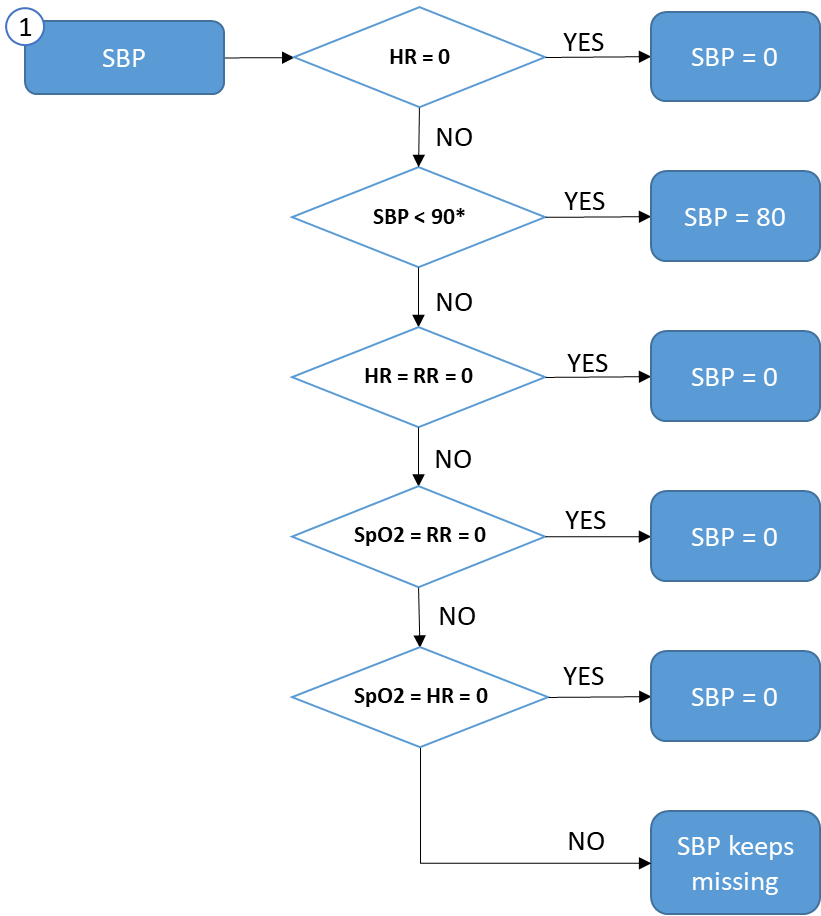


Supplementary Figure 1. Flowchart of SBP imputation based on clinical criteria. *Conditions with this symbol were extracted from TCC.

1. To impute HR when it was missing, the flowchart shown in Supplementary Figure 2 was carried out. The same way that SBP was imputed to zero when HR was zero, HR was considered zero when SBP was zero. In addition, if two of the other vitals (SBP, RR, or SpO2) were zero, HR was imputed to 0 bpm. If none of the above conditions were satisfied, the HR remained missing.


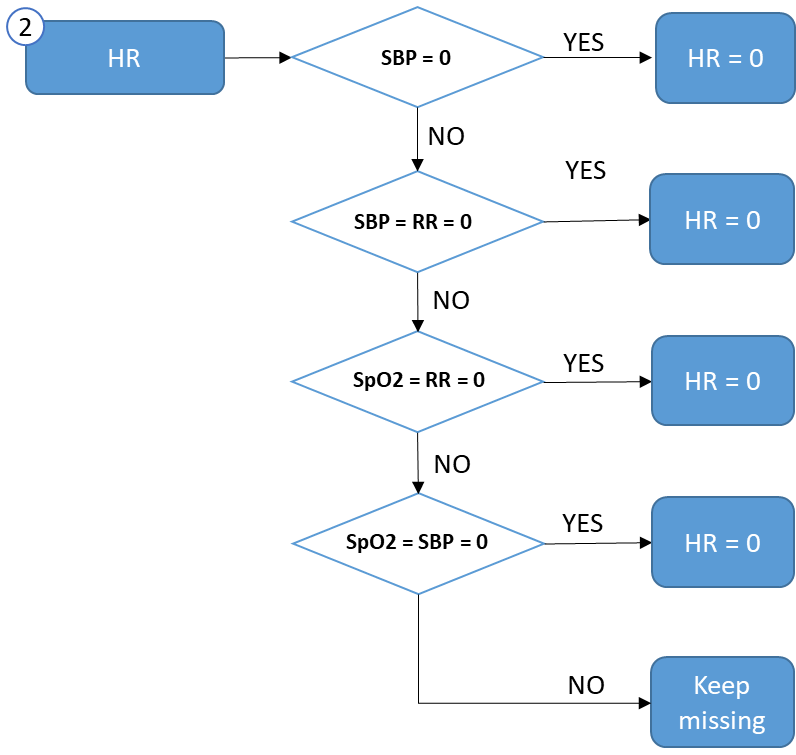


Supplementary Figure 2. Flowchart of HR imputation based on clinical criteria.

1. To impute RR the flowchart presented in Supplementary Figure 3 was carried out. TCC field can contain the ‘RR lower than 10 or higher than 29 breaths per minute’ condition. In that case, if other vitals were zero, then RR was also set to 0 breaths per minute, otherwise RR was set to 30 breaths per minute. In addition if two values out of SpO2, HR or SBP were zero, RR was completed with 0 breaths per minute. Finally, if none of the above conditions were satisfied, the RR remained missing.


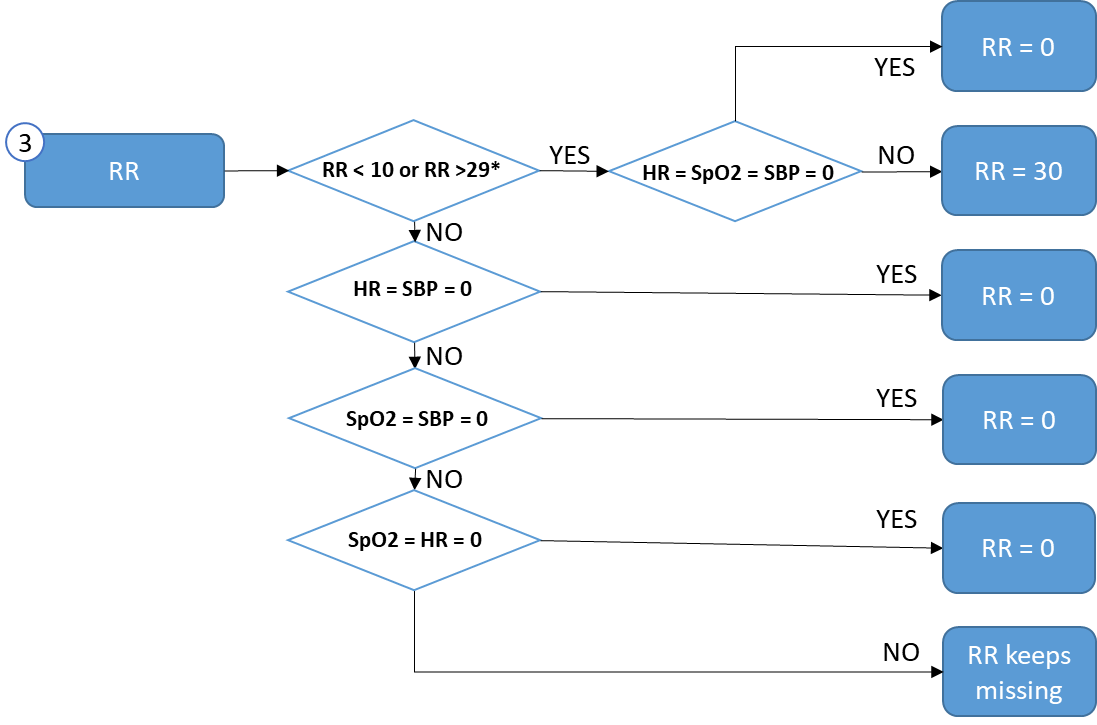


Supplementary Figure 3. Flowchart of RR imputation based on clinical criteria. *Conditions with this symbol were extracted from TCC.

1. GCS was imputed following the flowchart presented in Supplementary Figure 4. AVPU field was first checked and, depending on its value, GCS was fixed to 15 when AVPU was ‘alert’, to 13 when ‘verbal’, to 8 when ‘painful’, or to 3 when ‘unconscious’. If AVPU was also missing, TCC could contain a code with the condition Glasgow Coma Score less than or equal to 13. In that case, if the vitals were 0, then GCS was imputed to 3, otherwise to 13.


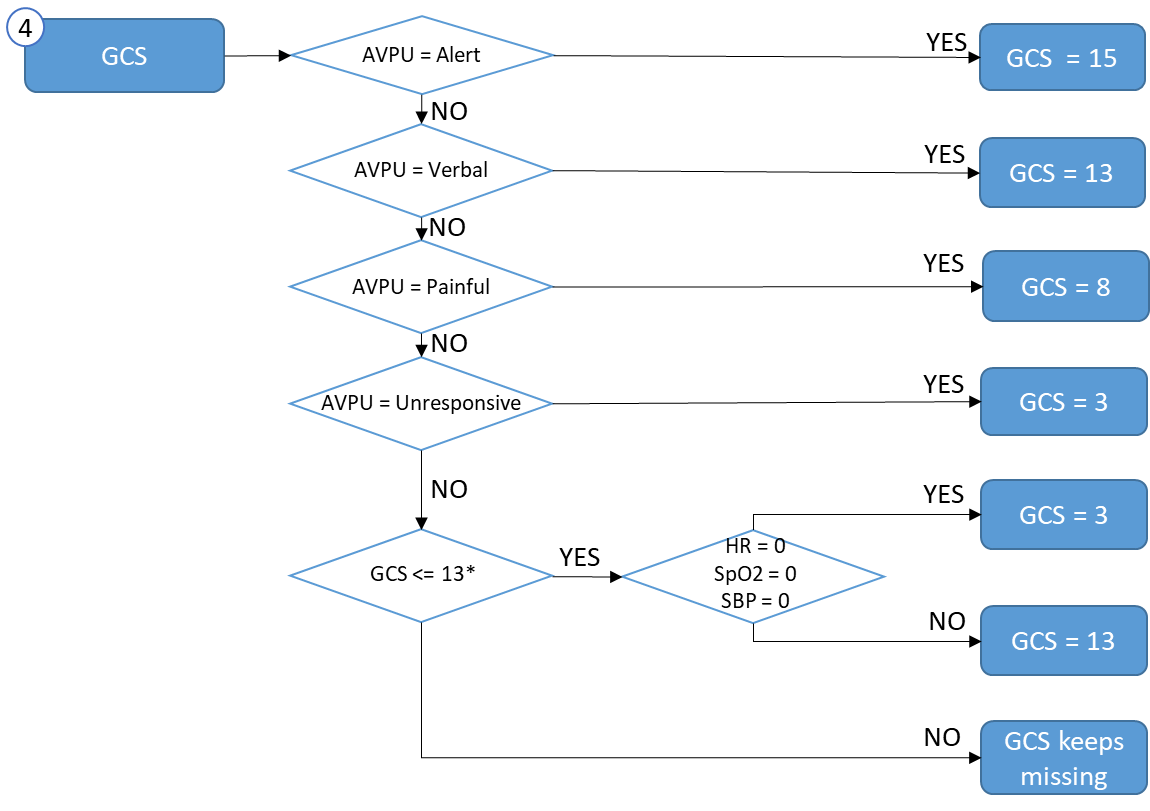


Supplementary Figure 4. Flowchart of GCS imputation based on clinical criteria. *Conditions with this symbol were extracted from TCC.

1. SpO2 was imputed following flowchart presented in Supplementary Figure 5. If two vitals out of HR, RR or SBP were zero, SpO2 was set to 0%. Otherwise, the SpO2 remained missing.


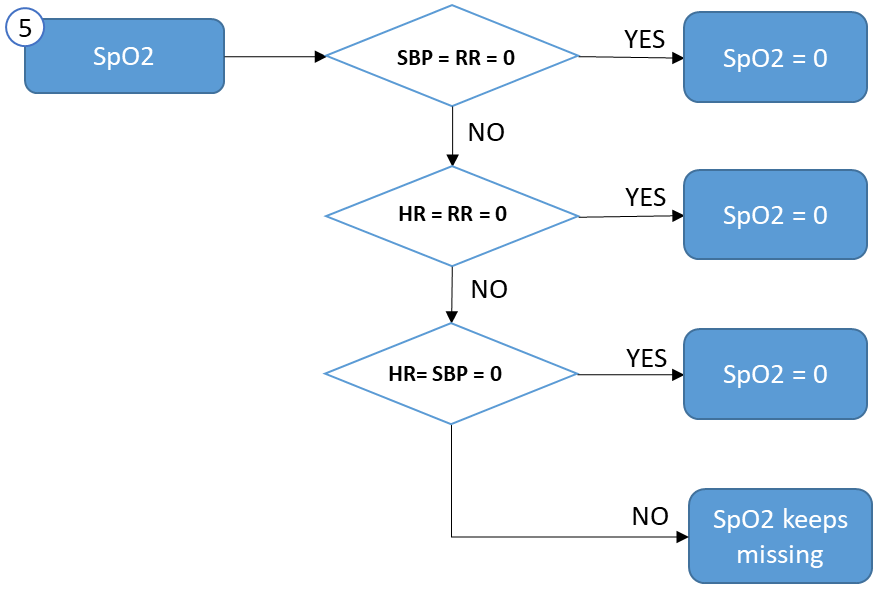


Supplementary Figure 5. Flowchart of SpO2 imputation based on clinical criteria.

**Machine learning based imputation**

The second method used for vital sign imputation in the NEMSIS dataset was a non-parametric machine learning method, namely the *K*-Nearest Neighbors (KNN). Concretely, the method was applied with *K*=10 to search for the *K* cases with most ‘similar’ vitals (‘neighbors’) to those present in the case to be imputed. Similarity was measured as proximity in terms of Euclidean distance, $d_{E}$, as described in equation 1.

$d_{E}(\boldsymbol{p},\boldsymbol{q}) =\sqrt{\sum_{i=1}^{n} \left( p_{i}- q_{i} \right)^{2}}$ (1)

where the Euclidean distance, $d_{E}(\boldsymbol{p},\boldsymbol{q})$, between the case $\boldsymbol{p}$ to be imputed and another case $\boldsymbol{q}$ containing all the four vitals is computed. Variables $p_{i}$ and $q_{i}$ represent the *i*^th^ vital out of those $n$ vitals available in the case to be imputed.

The nearest neighbor corresponds to that case $\boldsymbol{x}$ with shortest Euclidian distance to case $\boldsymbol{p}$ to be imputed. The *K* nearest neighbors are then identified and used to impute missing variable $w$ of case $\boldsymbol{p}$, $w^{(p)}$, as the average value of variable $w$ along the *K* nearest neighbors as described in equation 2.

$w^{(p)}= \frac{1}{K}\sum_{j=1}^{K} w^{(j)}$ (2)
